# Association of a CD44s-v5-v6 Null Phenotype with Advanced Stage Cholangiocarcinoma: A Preliminary Study

**DOI:** 10.3390/cancers18010021

**Published:** 2025-12-20

**Authors:** Kyaw Zwar Myint, Thanakrit Mongkonsiri, Artit Jinawath, Rutaiwan Tohtong

**Affiliations:** 1 Department of Biochemistry, Faculty of Science, Mahidol University, Bangkok 10400, Thailand; kyaw.zwa@mahidol.ac.th; 2 Department of Pathology, Faculty of Medicine, Ramathibodi Hospital, Mahidol University, Bangkok 10400, Thailand; narongsak.mog@mahidol.ac.th

**Keywords:** cholangiocarcinoma, CD44, CD44 isoforms, prognostic biomarkers

## Abstract

**Background/Objectives**: Cholangiocarcinoma (CCA) is an aggressive malignancy with a poor prognosis, creating an urgent need for novel biomarkers to improve risk stratification. The prognostic significance of the transmembrane glycoprotein CD44 and its isoforms (CD44s, v5, v6) in CCA remains controversial. This preliminary study aimed to investigate whether the combined loss of these isoforms could serve as a distinct prognostic indicator. **Methods**: We evaluated the expression of CD44s, CD44v5, and CD44v6 via immunohistochemistry on a retrospective cohort of 61 paraffin-embedded CCA patient tissue blocks from Ramathibodi Hospital, Bangkok, Thailand. Expression levels were correlated with clinicopathological parameters. Survival analyses, including Kaplan–Meier and Cox proportional hazards models, were used to determine the prognostic value of individual isoforms and the complete absence of all three. **Results**: Expression of CD44s, CD44v5, and CD44v6 was found in 52.5%, 47.5%, and 82.0% of tumors, respectively. In univariate and multivariate analyses, the expression of any single isoform was not a significant predictor of overall survival. However, a subgroup of 8 patients (13.1%) was identified whose tumors were negative for all three isoforms, a phenotype we termed “CD44s-v5-v6 Null”. This status was significantly associated with advanced TNM stages (*p* = 0.022). Patients with these Null tumors also showed a clinically relevant, though not statistically significant, trend towards poorer survival (median 7.0 vs. 12.0 months, *p* = 0.336). **Conclusions**: Individual CD44 isoforms did not serve as reliable independent prognostic markers in this cohort. Instead, the complete loss of the CD44 expression program characterizes a potential “CD44s-v5-v6 Null” phenotype associated with advanced-stage disease.

## Simple Summary

Cholangiocarcinoma is a very aggressive bile duct cancer with poor survival rates, making it crucial for doctors to better predict patient outcomes. Scientists have long studied proteins on the surface of cancer cells, called CD44, as potential clues, but their role has been unclear. This study investigated three specific CD44 proteins in patient tumors to see if their presence could predict the disease’s course. Unexpectedly, the researchers found that the tumors completely lacking all three of these proteins were associated with more advanced-stage cancer. This suggests that the absence of these markers, rather than their presence, could identify a new high-risk patient group. This finding may help doctors better classify tumors and could guide future research into new treatment approaches for these high-risk patients.

## 1. Introduction

Cholangiocarcinoma (CCA), a malignancy arising from the biliary epithelium, presents a formidable challenge in clinical oncology due to its aggressive behavior, limited therapeutic options, and rising global incidence [1,2,3,4,5]. Accounting for 15% of primary liver tumors, CCA is relatively rare worldwide but imposes a disproportionately high burden in regions like Thailand, where elevated mortality rates contribute to significant annual deaths [6,7]. The disease’s insidious onset and late-stage diagnosis often preclude curative surgical intervention, resulting in a dismal prognosis and 5-year survival rates below 20% [8,9,10,11]. Although progress has been made in elucidating the molecular pathogenesis of cholangiocarcinoma (CCA), effective methods for its early detection remain elusive. Therefore, identifying novel prognostic biomarkers is crucial for enhancing risk stratification and improving patient management [9,12].

CD44 is a transmembrane glycoprotein that exists in multiple isoforms due to alternative splicing, producing the standard form (CD44s) and various variant isoforms (CD44v), including CD44v5 and CD44v6. CD44s is broadly expressed in many cell types, while CD44v isoforms are more restricted and often upregulated in aggressive tumors [13,14]. Both CD44s and CD44v isoforms play crucial but sometimes overlapping or even contradictory roles in cancer, contributing to processes such as tumor initiation, proliferation, invasion, metastasis, epithelial–mesenchymal transition (EMT), stemness, and therapy resistance [15]. CD44v isoforms, in particular, can act as co-receptors for growth factors and cytokines, facilitating signaling pathways that promote tumor progression and metastasis. Notably, CD44v5 is linked to proliferative activity and growth in tumors, while CD44v6 is strongly associated with poor prognosis and metastatic potential in several cancers, including colorectal, pancreatic, and hepatocellular carcinomas [16,17].

In cholangiocarcinoma (CCA), immunohistochemical studies have shown that CD44s, CD44v5, and CD44v6 are frequently neoexpressed at the membrane of cancer cells, whereas they are absent in normal bile ducts. However, existing literature presents contradictory findings regarding the prognostic utility of these isoforms. Earlier studies suggested that the individual expression of these isoforms does not significantly correlate with tumor grade, metastasis, or vascular invasion, with the exception that aberrant CD44s expression was sometimes linked to the absence of metastasis and vascular invasion [18].

Conversely, a large immunohistochemical study by Padthaisong et al. (2020) [19] of 178 CCA samples provided contrasting evidence: they found that elevated CD44 and CD44v6 expression are significantly associated with shorter recurrence-free and overall survival. Specifically, CD44v6 overexpression correlated with higher tumor stage, and both CD44 and CD44v6 (as well as CD44v8-10) were linked to poor outcomes and increased risk of recurrence. This work further demonstrated that combining these markers with CA19-9 improved the prediction of recurrence in early-stage CCA [19].

These conflicting reports highlight a critical gap: the prognostic utility of individual CD44 isoforms in CCA remains unresolved. The inconsistent findings suggest that focusing on single markers may be insufficient and that the true biological and clinical significance could lie in more complex expression patterns. To date, most studies have focused on the presence or overexpression of specific variants. The prognostic value of the complete loss of the CD44 expression program—simultaneously negative for standard (s), v5, and v6 isoforms—has not been characterized in cholangiocarcinoma.

Therefore, this preliminary study aimed to investigate the combined expression patterns of CD44s, v5, and v6 in a Thai CCA cohort. Specifically, we hypothesized that the coordinated loss of these isoforms (a “CD44s-v5-v6 Null” phenotype) might define a biologically distinct and aggressive subset of tumors, distinct from those retaining CD44 expression.

## 2. Materials and Methods

### 2.1. Ethical Statement and Study Cohort

This study was conducted in accordance with the principles of the Declaration of Helsinki (1964) and its later amendments. Ethical approval (MURA2011/402) (Protocol Number ID 08-54-29) was obtained from the Ethical Committee of Ramathibodi Hospital, Bangkok, Thailand.

A retrospective cohort of 61 cholangiocarcinoma (CCA) cases was recruited from the Department of Pathology, Ramathibodi Hospital, spanning the years 2003–2015. The cohort consisted of 48 intrahepatic and 13 extrahepatic CCA types. All tumors were classified according to the American Joint Committee on Cancer and Union for International Cancer Control (AJCC/UICC) 7th edition. Histological grading was performed based on tumor morphology, classifying tumors as well, moderately, or poorly differentiated.

### 2.2. Immunohistochemistry (IHC)

#### 2.2.1. Tissue Preparation and Staining

Paraffin-embedded tissue blocks were sliced into 4 μm thick sections. Immunohistochemical analysis for CD44 standard form (CD44s) and variant isoforms CD44v5 and CD44v6 was performed using the Bond-Max automated immunostainer (Leica Microsystems, Newcastle, UK). The primary mouse monoclonal antibodies used in this study were as follows: CD44s (clone 156-3C11; Cell Signaling Technology^®^, Danvers, MA, USA) used at a 1:200 dilution; CD44v5 (clone VFF-8; eBioscience, Vienna, Austria) used at a 1:50 dilution; and CD44v6 (clone VFF-18; eBioscience, Austria) used at a 1:500 dilution.

#### 2.2.2. IHC Scoring and Evaluation

For each marker, staining was evaluated by a certified pathologist who was blinded to the clinical outcomes. Given the retrospective nature of the study and the use of archival tissues, a binary classification system was employed to minimize inter-observer variability associated with intensity grading. The expression of the CD44 proteins was considered positive if ≥10% of the tumor cells showed membranous, cytoplasmic, or luminal staining. Internal controls were verified for each slide: adjacent normal bile ducts served as internal negative controls, while stromal inflammatory cells (lymphocytes/macrophages) served as internal positive controls. Stained sections were visualized and captured using an Olympus BX53 microscope (Olympus Corporation, Tokyo, Japan).

### 2.3. Statistical Analysis

Data analysis was performed using IBM SPSS Statistics version 30.0.0.0 (SPSS, Chicago, IL, USA). Associations between clinicopathological features and CD44s, CD44v5, and CD44v6 expression were analyzed by Chi-square or Fisher’s exact test. Survival was calculated using the Kaplan–Meier method. Univariate and Multivariate Cox proportional hazard models were used to identify the statistically independent factors. A *p*-value < 0.05 was considered significant.

### 2.4. AI-Assisted Manuscript Preparation

During the preparation of this manuscript, the authors utilized Gemini 3.0 Pro to enhance readability and to build the tables from analysis files. The authors reviewed and edited the manuscript as necessary and assume full responsibility for the content of the publication.

## 3. Results

### 3.1. Patient Cohort and CD44 Isoform Expression

**Figure 1:**
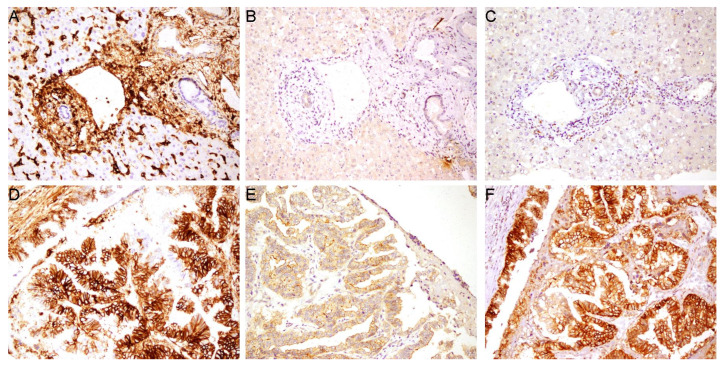
Immunohistochemical staining of CD44 isoforms in cholangiocarcinoma and adjacent normal tissue. Representative images show the expression of (**A**) CD44s, (**B**) CD44v5, and (**C**) CD44v6 in tumor-adjacent normal bile ducts and (**D**) CD44s, (**E**) CD44v5, and (**F**) CD44v6 in cholangiocarcinoma tissues. 20× magnification.

A total of 61 patients with cholangiocarcinoma (CCA) were included in this study. The expression of CD44 standard (CD44s) and variant isoforms (CD44v5, CD44v6) was evaluated by immunohistochemistry (IHC). In tumor-adjacent normal tissue, the bile ducts themselves showed minimal or absent expression for all three isoforms (Figure 1A–C). Strong CD44s staining was observed in the tissue surrounding the normal bile ducts, which served as an internal positive control confirming antibody specificity (Figure 1A); however, the adjacent liver parenchyma showed minimal to no staining for CD44v5 and CD44v6 (Figure 1B,C). In contrast, CCA tissues demonstrated variable expression patterns, with positive staining observed predominantly on the cell membrane of tumor cells (Figure 1D–F). Overall, positive expression for CD44s, CD44v5, and CD44v6 was observed in 32 (52.5%), 29 (47.5%), and 50 (82.0%) of the 61 tumor samples, respectively.

### 3.2. Association of CD44 Isoform Expression with Clinicopathological Features

We next investigated the association between the expression of each CD44 isoform and various clinicopathological parameters (Table 1). Positive expression of CD44s was significantly associated with intrahepatic tumor location (*p* = 0.050), multiple tumor focality (*p* = 0.037), and poor histological differentiation (*p* = 0.005). Expression of CD44v6 was significantly associated with earlier TNM stages (Stage I/II) (*p* = 0.018). No other significant associations were found between CD44s, CD44v5, or CD44v6 expression and parameters such as patient sex, age, tumor size, or lymphovascular invasion.

### 3.3. Univariate and Multivariate Survival Analyses

**Figure 2:**
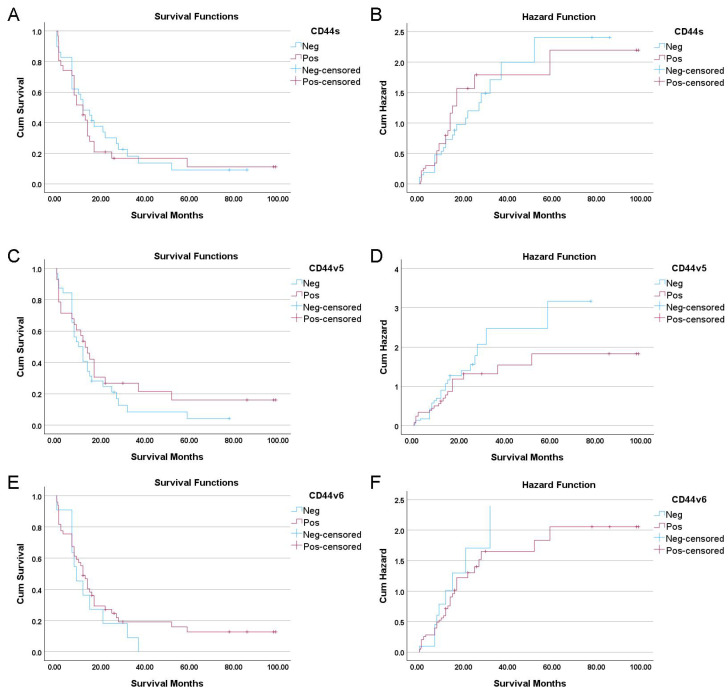
Kaplan–Meier survival and cumulative hazard curves for CD44 isoform expression in cholangiocarcinoma patients. Survival curves (**left panel**) and cumulative hazard functions (**right panel**) are shown for (**A**,**B**) CD44s, (**C**,**D**) CD44v5, and (**E**,**F**) CD44v6 expression. No statistically significant differences in overall survival were observed for any individual isoform.

Further, to identify factors associated with overall survival, a univariate Cox regression analysis was performed. Several established clinical features were significant predictors of poor outcomes: mass-forming macroscopic growth type (HR = 2.3, 95% CI: 1.27–4.17, *p* = 0.006), higher histological grade (*p* < 0.001), larger tumor size (≥5cm; HR = 1.89, 95% CI: 1.01–3.54, *p* = 0.046), presence of lymph node metastasis (HR = 1.87, 95% CI: 1.06–3.30, *p* = 0.031), and advanced TNM stage (III/IV; HR = 1.88, 95% CI: 1.07–3.28, *p* = 0.028) (Table 2). In stark contrast, the expression status of the individual biomarkers was not associated with patient outcomes. Kaplan–Meier analysis showed no significant association between the expression of individual CD44 isoforms and overall survival (Figure 2). This was confirmed in the univariate Cox regression, where expression of CD44s (HR = 1.15, *p* = 0.630), CD44v5 (HR = 0.76, *p* = 0.333), or CD44v6 (HR = 0.74, *p* = 0.386) did not predict patient survival (Table 2).

### 3.4. Association Among CD44 Isoform Expression

Given that the expression of individual isoforms did not predict survival, we next investigated their co-expression patterns. This analysis was performed to determine if the isoforms are regulated in a coordinated manner, which could reveal more complex biological relationships and suggest that their combined expression status, rather than their individual presence, might hold greater clinical significance.

Analysis of co-expression patterns revealed that the expression of CD44s and CD44v5 was not significantly associated with each other (Phi = 0.117, *p* = 0.359). However, a significant positive association was found between the expression of CD44s and CD44v6 (Phi = 0.322, *p* = 0.012), and an even stronger positive association was observed between CD44v5 and CD44v6 (Phi = 0.361, *p* = 0.005) (Table 3).

### 3.5. Multivariate Survival Analysis

Having established that individual CD44 isoforms were not prognostic in univariate analysis, the next critical step was to determine if they held any predictive power independent of established clinicopathological risk factors. Therefore, we constructed a multivariate Cox proportional hazards model to assess whether the expression of CD44s, CD44v5, or CD44v6 could provide additional prognostic information beyond that of the significant clinical variables identified previously.

First, a baseline clinical model was established using the significant predictors from the univariate analysis. In this initial model, only poor histological differentiation remained a strong and independent predictor of poor survival (HR = 3.47, *p* = 0.015), while other factors like TNM stage and lymph node metastasis lost their significance after adjustment.

Subsequently, each CD44 isoform was individually added to this baseline model to test its independent prognostic value. The addition of CD44s expression did not contribute significantly to the model (HR = 0.77, *p* = 0.484) and did not alter the primary finding that poor histology was the key predictor. Similarly, CD44v5 status was not an independent predictor of survival (HR = 0.80, *p* = 0.507) when adjusted for the clinical variables. Finally, adding CD44v6 also showed no independent prognostic significance (HR = 0.60, *p* = 0.189).

In all adjusted models, poor histology consistently emerged as the sole significant independent factor, confirming that the CD44 isoforms do not offer additional prognostic value when key clinical variables are considered (Table 4).

### 3.6. Prognostic Significance of a “CD44s-v5-v6 Null” Phenotype

The lack of individual prognostic significance for any single CD44 isoform, combined with their strong pattern of co-expression, suggested that the true prognostic signal might not lie in a specific isoform but rather in the status of the entire CD44 expression program. Based on this rationale, we hypothesized that the complete absence of these markers—a phenotype we termed “CD44s-v5-v6 Null”—defines a biologically distinct tumor subtype. To investigate this, we stratified the patient cohort into two groups, namely a “CD44s-v5-v6 Null” group (*n =* 8), whose tumors lacked expression of all three isoforms, and a “CD44s-v5-v6+” group (*n =* 53), whose tumors expressed at least one isoform, and subsequently compared their clinical outcomes.

Despite a comprehensive comparison across numerous clinicopathological variables, the only significant difference found was in the TNM stage. Specifically, CD44s-v5-v6 Null status was significantly associated with a more advanced TNM stage (III/IV) at the time of diagnosis (*p* = 0.022). This finding is particularly striking because other critical markers of tumor aggressiveness—such as poor histological grade, lymphovascular invasion, and lymph node metastasis—did not show a significant association with the pan-negative status. This suggests a specific link between the loss of the CD44 expression program and the overall tumor stage, a key determinant of prognosis (Table 5).

**Figure 3:**
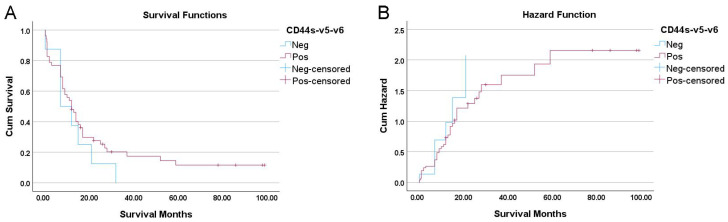
Kaplan–Meier survival and cumulative hazard curves for CD44s-v5-v6 status: (**A**) Survival curves and (**B**) cumulative hazard functions for patients stratified by pan-negative versus CD44-positive status. The difference in survival was not statistically significant (log-rank *p* = 0.336).

The survival analysis further highlighted the potential clinical importance of this pan-negative phenotype. Kaplan–Meier analysis revealed a clinically notable trend toward worse outcomes for patients in the “CD44s-v5-v6 Null” group. These patients had a median overall survival of just 7.0 months, compared to 12.0 months for patients whose tumors expressed at least one CD44 isoform. This five-month difference in median survival, while substantial from a clinical perspective, did not achieve statistical significance as determined by the log-rank test (*p* = 0.336) (Figure 3).

Furthermore, the univariate Cox regression analysis showed a hazard ratio (HR) of 1.45 for the pan-negative group. This indicates that these patients had a 45% higher risk of death compared to the CD44-positive group, but this finding was not statistically significant (*p* = 0.336). To determine if this survival trend held independent of confounding factors, we performed a multivariate Cox regression adjusting for histological grade, TNM stage, lymph node metastasis, and tumor size. In this adjusted model (Table 4), the CD44s-v5-v6 Positive status (versus Negative) exhibited a hazard ratio of 0.602 (95% CI: 0.252–1.438). This indicates that patients retaining CD44 expression had a reduced risk of death compared to the Null group (a protective trend), although this did not reach statistical significance (*p* = 0.253) due to the limited sample size.

## 4. Discussion

In this study, we investigated the prognostic significance of CD44 standard and variant isoforms in a cohort of 61 cholangiocarcinoma patients. Our findings demonstrate that while these isoforms are frequently expressed in CCA, the expression level of any single isoform—CD44s, CD44v5, or CD44v6—was not independently associated with overall survival. This result contributes to a landscape of conflicting literature. While some studies, such as the large analysis by Padthaisong et al., have linked high expression of CD44 and its variant CD44v6 to poor prognosis and recurrence in CCA [19], our findings align more closely with other reports suggesting a lack of independent prognostic value for individual isoforms [18]. This discordance underscores the complex and context-dependent role of CD44 in CCA progression.

First, we identified a significant link between CD44s expression and features of aggressive disease, including poor histological differentiation, multifocality, and intrahepatic location. This finding is noteworthy because it presents a biological paradox. CD44s is often associated with maintaining normal epithelial integrity, and its loss can be a marker of aggressive epithelial–mesenchymal transition (EMT) in other cancers [20]. Our results suggest a more complex, context-dependent role in cholangiocarcinoma, where CD44s expression may be co-opted by a subset of aggressive tumors. This subtype might rely on CD44s-mediated cell adhesion or signaling to facilitate multifocal growth and invasion through mechanisms distinct from a complete EMT. However, the crucial observation was that this association with aggressive pathology did not translate into a survival disadvantage. This disconnect implies that while CD44s may be a marker of a particular aggressive growth pattern, it is not the ultimate driver of mortality, prompting us to search for a more robust prognostic signal.

Second, the observation of strong co-expression patterns, particularly the significant positive correlation between CD44v6 and the other two isoforms, was highly informative. This suggests that the expression of these isoforms is not random but is likely part of a coordinated biological program, potentially driven by common upstream transcriptional or splicing factors activated in cancer cells. The co-expression of CD44v6, a known co-receptor for growth factors that promotes metastasis, alongside CD44s and CD44v5, points toward a potential functional synergy. This led us to hypothesize that the true prognostic information may not reside in the overexpression of any single isoform, which could represent just one component of a larger mechanism, but rather in the overall status of the entire CD44 expression program.

The most significant finding of this study is the identification of a potential high-risk subgroup characterized by a “CD44s-v5-v6 Null” phenotype—the complete absence of CD44s, CD44v5, and CD44v6 expression. This subgroup was significantly associated with a more advanced TNM stage at diagnosis. Our multivariate analysis indicated that this trend persisted even after adjusting for TNM stage and histological grade. In the adjusted model, the CD44-positive status yielded an HR of 0.602 (indicating a protective benefit compared to the Null phenotype). This suggests that the poor prognosis associated with the Null phenotype is not merely a consequence of advanced tumor stage, but potentially reflects an intrinsic aggressive biology independent of tumor burden. This novel finding suggests that the loss of the CD44 program may signify a distinct, aggressive biological subtype of CCA that relies on alternative oncogenic pathways for its progression.

The role of CD44 in cancer is complex and often appears contradictory. While it is widely recognized as a cancer stem cell marker whose overexpression promotes tumor progression, invasion, and metastasis in many cancers, a growing body of evidence reveals it can also function as a tumor suppressor [15]. This suppressive role is primarily linked to the standard isoform, CD44s, which is crucial for maintaining epithelial integrity and polarity. The loss of CD44s has been associated with epithelial–mesenchymal transition (EMT) and, paradoxically, with more aggressive disease in specific contexts [20]. For example, in breast cancer, the loss of CD44s correlates with more aggressive tumors and poorer survival [15]. Similarly, reduced CD44 expression in colorectal cancer is linked to deeper tumor invasion and lymph node metastasis [21]. This dual functionality highlights that the clinical significance of CD44 is highly context-dependent, where its loss, rather than its presence, can be a key indicator of aggressive tumor behavior leading to poor patient survival.

The concept that a loss of CD44 expression, rather than its overexpression, could signify a more aggressive phenotype is supported by evidence from a large study of 410 primary urothelial bladder cancers, which found that absent CD44v6 expression was an independent predictor of poor outcome. Specifically, tumors lacking CD44v6 had a higher grade, more advanced stage, 2.3-fold increased risk of recurrence, and significantly shorter median overall survival (30 vs. 75 months) compared to those with CD44v6 expression. Multivariate analysis confirmed that loss of CD44v6 was an adverse prognostic factor for both recurrence and overall survival in bladder cancers [22]. Another long-term follow-up in bladder cancers study showed that strong CD44v6 expression in tumor cells was associated with higher survival probability, and that loss of CD44v6 correlated with higher grade and more aggressive tumor features [23].

The underlying mechanism for this paradoxical effect may relate to the disruption of CD44’s normal function in maintaining cell–cell adhesion; its loss could facilitate an epithelial–mesenchymal transition (EMT), thereby promoting tumor cell invasion and dissemination [13,24]. This counterintuitive role, where loss of expression is detrimental, highlights the context-dependent nature of CD44 in cancer biology and lends external support to our hypothesis that the CD44s-v5-v6 Null phenotype in CCA may represent a distinct and highly aggressive disease subtype.

However, several limitations must be acknowledged when interpreting these results. The primary limitation is the retrospective nature of the study and the small sample size, particularly of the pan-negative group (*n =* 8). This small subgroup size likely lacked the statistical power required to detect a significant difference in survival, despite the observed trend. Additionally, as a single-center study, the findings may have limited generalizability. Additionally, due to the use of archival tissues, we employed a binary scoring system (≥10%) to ensure reproducibility; however, future prospective studies should employ composite scoring systems (such as H-scores) to evaluate whether staining intensity offers additional prognostic granularity. Finally, we were unable to perform molecular validation (e.g., RT-qPCR or Western blotting) due to the quality of the archival specimens. Future studies using fresh tissue are warranted to investigate the expression of EMT markers (such as E-cadherin and Vimentin) and upstream splicing regulators to mechanistically validate the ‘Null’ phenotype.

In conclusion, this preliminary study challenges the conventional focus on individual CD44 isoform overexpression as a prognostic tool in CCA. Instead, it provides the first evidence that the complete loss of the CD44 expression program may identify a subset of patients with advanced disease and a tendency for worse outcomes. This “CD44s-v5-v6 Null” phenotype represents a novel and potentially crucial putative biomarker that warrants validation in larger, multi-center prospective cohorts.

## 5. Conclusions

In conclusion, this preliminary study demonstrates that while individual CD44 isoforms are not independent prognostic markers in cholangiocarcinoma, the complete absence of CD44s, v5, and v6 expression characterizes a potential “CD44s-v5-v6 Null” subtype. This triple-negative status is significantly associated with advanced TNM stage and shows a clinically relevant, albeit not statistically significant, trend towards poorer overall survival that persisted in multivariate adjustment. This finding offers an alternative perspective to the conventional focus on isoform overexpression and suggests that the loss of the CD44 expression program may identify a distinct, high-risk biological subtype of CCA. Given the limitations of sample size and retrospective design, this CD44s-v5-v6 Null signature represents a putative biomarker. Future validation in larger, multi-center cohorts using fresh tissue is required to confirm its prognostic value and to elucidate the associated molecular mechanisms, such as EMT markers and splicing regulators.

## Figures and Tables

**Table 1:** Clinicopathological characteristics of CCA patients in correlation with CD44 isoforms.

Variable	*n* (61)	CD44s			CD44v5			CD44v6		
		Neg	Pos	*p*-Value	Neg	Pos	*p*-Value	Neg	Pos	*p*-Value
Sex										
Male	38	20	18	0.306^a^	20	18	0.972 ^a^	7	31	1.000 ^b^
Female	23	9	14		12	11		4	19	
Age (year)										
≤60	33	13	20	0.167 ^a^	14	19	0.088 ^a^	4	29	0.317 ^b^
>60	28	16	12		18	10		7	21	
Macroscopic tumor growth										
Mass forming type	37	17	20	0.757 ^a^	20	17	0.757 ^a^	5	32	0.254 ^a^
Intraductal type	24	12	12		12	12		6	18	
Size										
<5 cm	21	10	11	0.640 ^a,Ψ^	13	8	0.200 ^a,Ψ^	5	16	0.579 ^a,Ψ^
≥5 cm	34	14	20		15	19		6	28	
Location										
Intrahepatic	48	19	29	0.050 ^b,^*^,§^	23	25	0.337 ^b,§^	9	39	1.000 ^b,§^
Extrahepatic	12	9	3		8	4		2	10	
Tumor focality										
Solitary	40	21	19	0.037 ^b,^*^,Ψ^	22	18	0.322 ^a,Ψ^	10	30	0.255 ^b,Ψ^
Multiple	15	3	12		6	9		1	14	
Histologic grade										
Well differentiated	36	21	15	0.005 ^c,^*	18	18	0.515 ^c^	7	29	0.634 ^c^
Moderately differentiated	16	8	8		8	8		3	13	
Poorly differentiated	9	0	9		6	3		1	8	
Lymphovascular invasion										
Yes	38	16	22	0.275 ^a^	21	17	0.573 ^a^	5	33	0.203 ^a^
No	23	13	10		11	12		6	17	
Lymph node metastasis										
Yes	33	15	18	0.723 ^a^	14	19	0.088 ^a^	4	29	0.317 ^b^
No	28	14	14		18	10		7	21	
Distant metastasis										
Yes	56	28	28	0.357 ^b^	30	26	0.662 ^b^	10	46	1.000 ^b^
No	5	1	4		2	3		1	4	
Perineural invasion										
Yes	44	19	25	0.273 ^a^	21	23	0.234 ^a^	8	36	1.000 ^b^
No	17	10	7		11	6		3	14	
Resected margin										
Free margin	34	13	21	0.134 ^a,§^	16	18	0.414 ^a,§^	8	26	0.320 ^b,§^
Not free margin	26	15	11		15	11		3	23	
TNM Staging										
Stage I, II	32	14	18	0.533 ^a^	14	18	0.152 ^a^	2	30	0.018 ^b,^*
Stage III, IV	29	15	14		18	11		9	20	

^a^ Pearson Chi-square, ^b^ Fisher’s exact test, ^c^ Goodman and Kruskal’s gamma, ^§^ missing (*n* = 1), ^Ψ^ missing (*n* = 6), * *p* < 0.05.

**Table 2:** Univariate analysis of factors predicting overall survival.

Variable	No. of Patients	Median OS (Months)	HR	(95% CI)	*p*-Value
Age					
≤60 years	32	11	1		0.307
>60 years	28	12	1.34	(0.77–2.33)	
Sex					
Male	38	11	1		0.326
Female	22	13	1.35	(0.74–2.43)	
Tumor Location					
Intrahepatic	47	12	1		0.940 ^§^
Extrahepatic	12	10	1.026	(0.523–2.01)	
Macroscopic Growth					
Intraductal	23	22	1		0.006 *
Mass forming	37	8	2.3	(1.27–4.17)	
Histology					<0.001 *
Well differentiated	35	15	1		
Mod vs. Well	16	7	2.22	(1.14–4.33)	0.019 *
Poor vs. Well	9	8	3.73	(1.65–8.42)	0.002 *
Tumor Size					
<5 cm	21	14	1		0.046 ^Ψ,^*
≥5 cm	33	9	1.89	(1.01–3.54)	
Tumor Focality					
Solitary	40	13	1		0.064 ^Ψ,^*
Multiple	14	3	1.9	(0.96–3.73)	
Lymph Node Metastasis					
No	32	14	1		0.031 *
Yes	28	8	1.87	(1.06–3.30)	
Distant Metastasis					
No	55	12	1		0.49
Yes	5	8	1.39	(0.55–3.51)	
Lymphovascular Invasion					
No	37	14	1		0.154
Yes	23	9	1.51	(0.86–2.67)	
Resected Margin					
Free margin	34	12	1		0.691
Not free margin	25	11	1.12	(0.64–1.98)	
Perineural Invasion					
No	43	12	1		0.359
Yes	17	12	1.33	(0.72–2.46)	
TNM Stage					
I, II	31	16	1		0.028 *
III, IV	29	8	1.88	(1.07–3.28)	
CD44s Expression					
Negative	29	12	1		0.630
Positive	31	12	1.15	(0.66–1.99)	
CD44v5 Expression					
Negative	32	10	1		0.333
Positive	28	13	0.76	(0.43–1.33)	
CD44v6 Expression					
Negative	11	9	1		0.386
Positive	49	12	0.74	(0.38–1.46)	

Hazard ratios (HRs) are presented to consistently reflect increased risk (HR ≥ 1.0). For variables where the original HR was <1.0 (i.e., protective), the value is inverted (HR′ = 1/HR), and the reference group is switched accordingly. ^§^ missing (*n* = 1), ^Ψ^ missing (*n* = 6), * *p* < 0.05, Wald test.

**Table 3:** Association between CD44 isoform expression.

Isoform Pair	Phi (Φ) Coefficient	*p*-Value
CD44s vs. CD44v5	0.117	0.359
CD44s vs. CD44v6	0.322	0.012 *
CD44v5 vs. CD44v6	0.361	0.005 *

* *p* < 0.05, Pearson Chi-square test; the Phi (Φ) coefficient.

**Table 4:** Multivariate analysis of factors predicting overall survival.

Variable	HR	95% CI	*p*-Value
Baseline Clinical Model			
Histology (Moderate vs. Well)	1.883	0.819–4.328	0.136
Histology (Poor vs. Well)	3.468	1.273–9.450	0.015 *
TNM Stage (III/IV vs. I/II)	0.993	0.399–2.473	0.988
Lymph Node Metastasis (Yes vs. No)	1.833	0.757–4.439	0.179
Macroscopic Growth (Intraductal vs. Mass)	0.874	0.365–2.094	0.763
Tumor Size (≥5 cm vs. <5 cm)	1.146	0.529–2.484	0.73
Model + CD44s			
Histology (Moderate vs. Well)	2.051	0.864–4.871	0.104
Histology (Poor vs. Well)	4.345	1.315–14.359	0.016 *
TNM Stage (III/IV vs. I/II)	0.918	0.356–2.365	0.859
Lymph Node Metastasis (Yes vs. No)	1.942	0.787–4.792	0.150
Macroscopic Growth (Intraductal vs. Mass)	0.899	0.378–2.138	0.809
Tumor Size (≥5 cm vs. <5 cm)	1.118	0.521–2.402	0.774
CD44s (Positive vs. Negative)	0.771	0.372–1.597	0.484
Model + CD44v5			
Histology (Moderate vs. Well)	1.945	0.850–4.451	0.115
Histology (Poor vs. Well)	3.355	1.226–9.180	0.018 *
TNM Stage (III/IV vs. I/II)	1.09	0.422–2.812	0.859
Lymph Node Metastasis (Yes vs. No)	1.612	0.622–4.174	0.325
Macroscopic Growth (Intraductal vs. Mass)	0.902	0.378–2.150	0.816
Tumor Size (≥5 cm vs. <5 cm)	1.228	0.556–2.715	0.611
CD44v5 (Positive vs. Negative)	0.797	0.407–1.560	0.507
Model + CD44v6			
Histology (Moderate vs. Well)	1.967	0.847–4.565	0.115
Histology (Poor vs. Well)	3.887	1.373–11.001	0.011 *
TNM Stage (III/IV vs. I/II)	0.86	0.336–2.199	0.752
Lymph Node Metastasis (Yes vs. No)	1.871	0.768–4.555	0.168
Macroscopic Growth (Intraductal vs. Mass)	0.817	0.331–2.021	0.662
Tumor Size (≥5 cm vs. <5 cm)	1.176	0.539–2.565	0.683
CD44v6 (Positive vs. Negative)	0.604	0.285–1.281	0.189
Model + CD44s-v5-v6			
Histology (Moderate vs. Well)	1.954	0.848–4.506	0.116
Histology (Poor vs. Well)	3.979	1.396–11.336	0.010 *
TNM Stage (III/IV vs. I/II)	0.897	0.353–2.276	0.819
Lymph Node Metastasis (Yes vs. No)	1.756	0.716–4.307	0.219
Macroscopic Growth (Intraductal vs. Mass)	0.855	0.349–2.094	0.733
Tumor Size (≥5 cm vs. <5 cm)	1.193	0.547–2.602	0.657
CD44s-v5-v6 (Positive vs. Negative)	0.602	0.252–1.438	0.253

* *p* < 0.05, Wald test.

**Table 5:** Clinicopathological characteristics of CCA patients in correlation with CD44s-v5-v6 status.

Variable	*n*	CD44s-v5-v6 Null	CD44s-v5-v6+	*p*-Value
Sex				
Male	38	4	34	0.461 ^a^
Female	23	4	19	
Age (year)				
≤60	33	3	30	0.451 ^a^
>60	28	5	23	
Macroscopic Tumor Growth				
Mass forming type	37	4	33	0.700 ^a^
Intraductal type	24	4	20	
Size				
<5 cm	21	4	17	0.464 ^a,Ψ^
≥5 cm	34	4	30	
Location				
Intrahepatic	48	7	41	1.000 ^a,§^
Extrahepatic	12	1	11	
Tumor Focality				
Solitary	40	7	33	0.423 ^a,Ψ^
Multiple	15	1	14	
Histologic Grade				
Well differentiated	36	5	31	0.402 ^b^
Moderately differentiated	16	3	13	
Poorly differentiated	9	0	9	
Lymphovascular Invasion				
No	38	4	34	0.461 ^a^
Yes	23	4	19	
Lymph Node Metastasis				
No	33	2	31	0.127 ^a^
Yes	28	6	22	
Distant Metastasis				
No	56	8	48	1.000 ^a^
Yes	5	0	5	
Perineural Invasion				
No	44	5	39	0.674 ^a^
Yes	17	3	14	
Resected Margin				
Free margin	34	6	28	0.446 ^a^
Not free margin	26	2	24	
TNM Stage				
I/II	32	1	31	0.022 ^a,^*
III/IV	29	7	22	

^a^ Fisher’s Exact Test, ^§^ missing (n = 1), ^Ψ^ missing (n = 6), * *p* < 0.05

## Data Availability

The data presented in this study are not publicly available due to ethical and privacy restrictions related to patient confidentiality. Data may be made available upon reasonable request from the corresponding authors, pending approval from the relevant institutional review board.

## Abbreviations

The following abbreviations are used in this manuscript:

CCA	Cholangiocarcinoma
CD44s	CD44 standard form
CD44v	CD44 variant isoforms
EMT	Epithelial–mesenchymal transition
HR	Hazard ratio
IHC	Immunohistochemistry
TNM	Tumor, Nodes, Metastasis (TNM staging)
